# Genomic Insights into Cutaneous Squamous Cell Carcinoma

**DOI:** 10.3390/cancers18040558

**Published:** 2026-02-09

**Authors:** Grace S. Saglimbeni, Tyson J Morris, Beau Hsia, Abubakar Tauseef

**Affiliations:** 1 School of Medicine, Creighton University, Phoenix, AZ 85012, USA; gracesaglimbeni@creighton.edu (G.S.S.); tysonmorris@creighton.edu (T.J.M.); beauhsia@creighton.edu (B.H.); 2 School of Medicine, Creighton University, Omaha, NE 68178, USA

**Keywords:** cutaneous squamous cell carcinoma, non-melanoma skin cancer, AACR Project GENIE, somatic mutations, *TP53*, *NOTCH1*, copy number alterations, biomarker discovery, cancer genomics

## Abstract

**Background**: Cutaneous squamous cell carcinoma (cSCC) represents one of the most common keratinocyte-derived malignancies encountered in clinical practice; however, its genomic landscape remains far less comprehensively characterized than that of other cutaneous cancers. This study aims to identify key molecular drivers and potential therapeutic targets by comprehensively characterizing the genomic landscape of cSCC using data from the American Association for Cancer Research (AACR) Project Genomics, Evidence, Neoplasia, Information, Exchange (GENIE) consortium. **Methods**: A retrospective cohort analysis of cSCC samples was performed utilizing AACR Project GENIE data accessed via the cBioPortal platform (v18.0-public) on 22 November 2025. Analyses included identification of recurrent somatic and copy-number alterations, pairwise gene–gene co-occurrence testing using Fisher’s exact tests with Benjamini–Hochberg False Discovery Rate (FDR) correction, and exploratory subgroup comparisons by sex and race, with statistical significance defined as *p* < 0.05. **Results**: Recurrent mutations were identified in *TP53* (83.5%), *NOTCH1* (56.3%), *KMT2D* (47.0%), *CDKN2A* (44.4%), *TERT* (41.4%), *ROS1* (34.3%), *FAT1* (33.3%), *NOTCH2* (31.2%), *ERBB4* (28.4%), and *KMT2A* (24.3%), reflecting disruption of the p53 pathway, cell-cycle control, Notch signaling, epigenetic regulation, telomere maintenance, RTK/MAPK pathways, and Wnt signaling. Statistically significant co-occurrence patterns were observed, and exploratory subgroup analyses evaluated mutation frequency differences by sex and race. **Conclusions**: This large, multi-institutional genomic analysis defines recurrent mutational and structural alterations in cSCC and highlights an integrated pattern of pathway disruption involving genomic integrity, differentiation, epigenetic control, and proliferative signaling. These findings enhance current understandings of the molecular architecture underlying this common yet genomically understudied malignancy and provide a foundation for future mechanistic studies and development of targeted diagnostic and therapeutic strategies.

## Simple Summary

Cutaneous squamous cell carcinoma (cSCC) is one of the most common non-melanoma skin cancers, yet its genomic landscape remains incompletely defined despite its clinical significance. Using the American Association for Cancer Research (AACR) Project Genomics Evidence Neoplasia Information Exchange (GENIE), this study explores the mutational profile of cSCC in a large, diverse patient cohort. We found frequent mutations in *TP53*, *NOTCH1*, *KMT2D*, *CDKN2A*, *TERT*, *ROS1*, *FAT1*, *NOTCH2*, *ERBB4*, and *KMT2A*, which disrupt pathways controlling DNA damage response, cell-cycle checkpoints, keratinocyte differentiation, chromatin regulation, telomere stability, growth factor signaling, and cell adhesion. Recurrent co-occurring alterations were identified, and exploratory subgroup analyses suggested possible race- and sex-based differences requiring further validation. These findings expand our understanding of the molecular features driving cSCC and provide a framework for future studies aimed at improving diagnostic and therapeutic strategies for patients with this common yet genomically understudied skin cancer.

## 1. Introduction

Cutaneous squamous cell carcinoma (cSCC) is one of the most common keratinocyte-derived skin cancers, accounting for a substantial proportion of non-melanoma skin cancer cases encountered in clinical practice and more than 1 million new cases per year [1]. cSCC arises from epidermal keratinocytes and is characterized histopathologically by invasive nests of atypical squamous cells, often with keratin pearls and varying degrees of keratinization [1,2]. While many tumors follow an indolent course, a subset progresses to locally invasive or metastatic disease, significantly increasing morbidity and mortality [1,3]. Advanced cSCC can spread to regional lymph nodes and distant organs, presenting considerable therapeutic and prognostic challenges.

cSCC incidence continues to rise worldwide, particularly among individuals with fair skin, hair, and eye color who have experienced extensive ultraviolet exposure [4,5]. It predominantly affects adults older than 65 years and is more common in men, with a male-to-female ratio of approximately 3:1 [1,6]. UV-induced disease occurs far less frequently in non-White populations and remains poorly quantified in individuals of mixed ethnic backgrounds. Nevertheless, keratinocyte carcinoma can arise in people of all races, ethnicities, and skin phototypes [6,7]. Most tumors arise on sun-exposed sites such as the head and neck, and precursor lesions, including actinic keratoses and Bowen’s disease, may progress to invasive carcinoma when left untreated [8]. Established risk factors include chronic ultraviolet radiation, immunosuppression, chronic wounds, and exposure to environmental carcinogens [6].

Although cSCC is frequently diagnosed in routine practice, early or poorly differentiated tumors can be difficult to recognize, as they may closely resemble benign keratotic or inflammatory lesions. While most cSCC is effectively treated with surgery, advanced and metastatic disease remains clinically challenging and motivates improved molecular characterization [1,3,6,9,10].

Past genomic studies have identified recurrent alterations commonly seen in *TP53*, *NOTCH1*, *NOTCH2*, *CDKN2A*, *PI3K*, *EGFR*, and cell-cycle pathways [1,11,12]. However, limited sequencing coverage and incomplete demographic representation have constrained prior efforts to define the full molecular landscape of cSCC. Variability in methodology and tumor heterogeneity further hindered comprehensive characterization and limited understanding of the genomic drivers underlying aggressive disease behavior, highlighting the need for broader molecular profiling [13].

This study leverages a large, harmonized, multi-institutional AACR Project GENIE cohort of 423 cSCC tumors to quantify recurrent somatic and copy-number alterations using consistent filtering criteria. In addition to reporting mutation frequencies, we integrate hotspot distributions, copy-number and loss of heterozygosity (LOH) events, and statistically tested gene–gene co-occurrence analyses within a single dataset. By consolidating these layers of genomic information, this analysis provides a standardized reference landscape of cSCC that reinforces established drivers such as *TP53*, *NOTCH1*, and *CDKN2A* while also quantifying additional recurrent alterations within a unified analytic framework.

## 2. Materials and Methods

This study was determined to be exempt from institutional review board oversight at Creighton University (Phoenix, AZ, USA), as it involved secondary analysis of de-identified, publicly accessible data from the AACR GENIE database. The dataset does not contain protected health information; therefore, individual patient consent was not required. All analyses were performed in accordance with institutional and ethical guidelines. Clinical and genomic data from 2017 onward were retrieved through cBioPortal (GENIE v18.0-public) on 22 November 2025.

Our patient cohort included a total of 406 patients contributing 423 cSCC tumor samples. Cases were selected by restricting the GENIE skin cancer, non-melanoma dataset to samples annotated as cSCC. Tumor specimens were classified as primary lesions, originating from the initial tumor site, or metastatic lesions, obtained from secondary anatomic locations. To assess differences in alteration frequencies per gene between primary and metastatic groups, chi-squared or Fisher’s exact testing was applied as appropriate based on expected cell counts. To minimize potential double-counting when multiple tumor specimens were available from a single patient, inferential analyses, including gene–gene co-occurrence and demographic enrichment testing, were conducted at the patient level using the unique GENIE patient identifier, with each patient counted once per analysis. Descriptive mutation frequencies are reported at the tumor-sample level.

AACR Project GENIE is a multi-institutional data-sharing consortium that aggregates clinically annotated tumor sequencing datasets generated across 19 participating cancer centers. In this cSCC cohort, cases were contributed by 16 centers; the distribution of samples and patients by center is provided in Appendix A Table A1.

All genomic data were generated using institution-specific targeted next-generation sequencing (NGS) assays rather than whole-exome or whole-genome platforms. Panel coverage varied across contributing institutions, with the majority of specimens sequenced using MSK-IMPACT468, MSK-IMPACT505, MSK-IMPACT410, and DFCI-ONCOPANEL-3.1. A detailed breakdown of sequencing assay identifiers, including sample- and patient-level counts, is provided in Appendix A Table A2. Differences in panel size and gene coverage were considered when interpreting alteration frequencies to mitigate platform-related variability and improve cross-assay comparability. Sequencing depth typically exceeded 500×, consistent with expectations for high-coverage targeted assays. Approximately 53% of samples originated from primary tumor sites, and all specimens were sequenced as tumor-only cases. Matched tumor–normal information was not annotated in the dataset, which limited the capacity to systematically distinguish potential germline variants from somatic alterations.

GENIE applies centralized harmonization through Genome Nexus because contributing sites use institution-specific variant-calling and annotation workflows. As a result, variants are standardized to consistent nomenclature and annotation conventions across centers, even though upstream software choices and versions may differ. Accordingly, some bioinformatic heterogeneity can persist across participating institutions. Although GENIE includes treatment and outcome fields for select tumor types, treatment-response and longitudinal outcome data were not available for cSCC.

Somatic variants were retrieved from the GENIE harmonized mutation annotation format (MAF) files, which provide cross-institution standardization of gene symbols and protein-level variant annotations. We retained nonsynonymous variants (missense, truncating, splice-site, and in-frame events) and excluded synonymous alterations. Variants were additionally filtered using a minimum VAF of 5% and sequencing depth of at least 100×.

The dataset included genomic data, histologic classifications, and corresponding patient demographics, including sex, age, and race. Samples with unknown metadata required for a specific sub-analysis (e.g., sex or race) were excluded only from that sub-analysis rather than removed from the overall cohort. Genes without established clinical relevance were typically excluded from panel designs, synonymous mutations were omitted, and structural rearrangements and gene fusions were not analyzed. Copy-number alterations (including amplifications and LOH events) were analyzed as provided in the GENIE dataset.

All statistical analyses were performed using R/R Studio (R Foundation for Statistical Computing, Boston, MA, USA, version 4.5.1), with a two-sided *p*-value < 0.05 considered statistically significant. Continuous variables are reported as mean ± standard deviation (SD), and categorical variables are summarized as counts and percentages. Associations between categorical variables were assessed using the chi-squared test or Fisher’s exact test, as appropriate. For continuous variables, data distribution was assessed prior to analysis; normally distributed variables were compared using a two-sided Student’s *t*-test, whereas non-normally distributed variables were analyzed using the Mann–Whitney U test. To control for multiple hypothesis testing, *p*-values were adjusted using the Benjamini–Hochberg false discovery rate (FDR) procedure, with statistical significance defined as q < 0.05. Gene–gene co-occurrence and mutual exclusivity were evaluated using pairwise Fisher’s exact tests applied to 2 × 2 contingency tables. Effect sizes are reported as log2 odds ratios with corresponding 95% confidence intervals. The Clopper–Pearson exact binomial method was used to derive confidence intervals for co-occurrence proportions.

## 3. Results

### 3.1. Cohort Composition and Clinical Features

A total of 406 patients contributed 423 tumor samples to the cSCC dataset. In this cohort, 307 (75.6%) patients were male and 86 (21.2%) were female. Nearly all individuals were adults (99.8%), with only a single pediatric case. Ethnicity data revealed that 330 individuals (81.3%) were non-Hispanic/non-Spanish, 10 (2.5%) were Hispanic or Spanish, and 66 (16.3%) patients had no reported ethnicity. With respect to race, 347 (85.5%) patients were White, 6 (1.5%) were Black, 4 (1.0%) were Asian, 9 (2.2%) identified with other racial backgrounds, and 40 (9.9%) lacked racial annotation. Of the 423 tumor samples, 226 (53.4%) originated from primary lesions, 180 (42.6%) from metastatic sites, and 17 (4.0%) were unspecified. These demographic and sample features are summarized in Table 1.

### 3.2. Somatic Alterations in cSCC

**Figure 1:**
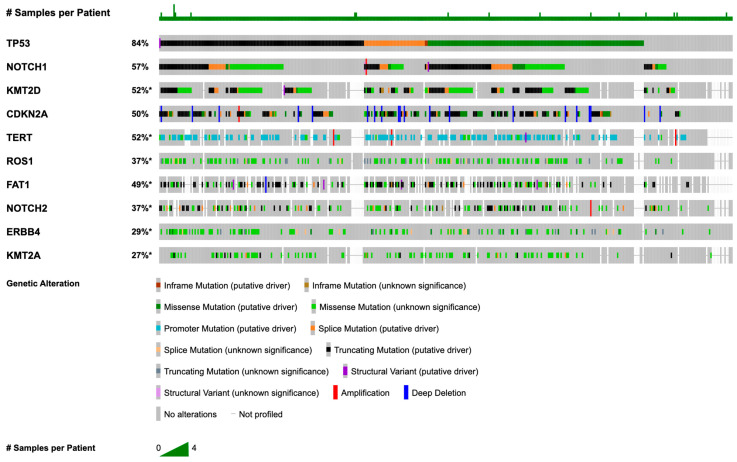
OncoPrint summarizing recurrent cSCC alterations (genes with *n* ≥ 5, depth ≥ 100×, VAF ≥ 5%). The asterisk (*) indicates that not all tumors were assayed for *KMT2D*, *TERT*, *ROS1*, *FAT1*, *NOTCH2*, *ERBB4*, and *KMT2A*. Percentages shown in the OncoPrint may differ slightly from Table 2 because the figure displays only samples with complete profiling across all listed genes.

Figure 1 summarizes the most frequent somatic alterations observed in this cSCC cohort, and corresponding mutation frequencies are listed in Table 2. The most frequently mutated gene was *TP53* (83.5%), followed by *NOTCH1* (56.3%), *KMT2D* (47.0%), *CDKN2A* (44.4%), *TERT* (41.4%), *ROS1* (34.3%), *FAT1* (33.3%), *NOTCH2* (31.2%), *ERBB4* (28.4%), and *KMT2A* (24.3%).

In addition to somatic mutations, we identified recurrent copy number alterations (CNAs) in 307 samples. LOH events were prevalent, particularly affecting tumor suppressor genes such as *CDKN2A* and *CDKN2B* (n = 20; 6.5% for all). Amplifications were also frequently observed in *CCND1* (n = 13; 4.2%) and *EGFR* (n = 10; 3.7%).

### 3.3. Characterization of Frequent Genomic Events

**Figure 2:**
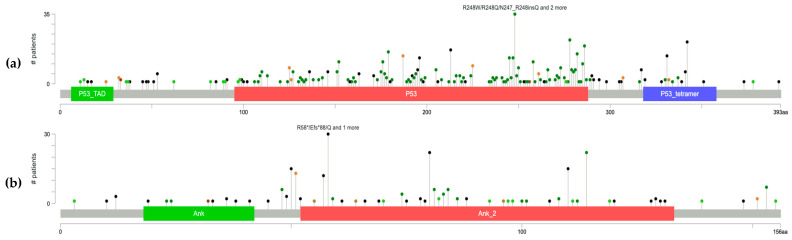
Lollipop plot of recurrent alterations in cSCC. (**a**) *TP53* and (**b**) *CDKN2A*.

Further analysis characterized the types of mutations among the most commonly altered genes. In *TP53*, alterations were predominantly missense with additional truncating events, including 33 cases affecting amino acid position R248 (R248W, R248Q, R248G, R248H, and N247_R248insQ), demonstrating clear recurrent hotspot activity as illustrated by the lollipop plot (Figure 2a). *NOTCH1* mutations were predominantly truncating with additional splice variants, and alterations were broadly distributed along the gene without evidence of focal clustering. *KMT2D* mutations followed a similar pattern, consisting mainly of truncating and splice alterations diffusely distributed along the gene. In *CDKN2A*, mutations were primarily truncating with some missense events, with 29 alterations involving the R58/V59 region (R58*, R58Efs*88, R58Q, V59del), highlighting a recurrent hotspot cluster critical for CDK4/6 interaction (Figure 2b). *TERT* mutations were primarily classified as other alteration types and were dispersed without identifiable hotspot formation. *ROS1* mutations were predominantly missense with some truncating events, and alterations were scattered throughout the gene without recurrent focal changes. *FAT1* mutations included both missense and truncating alterations, with variants distributed across the gene and no hotspot localization. *NOTCH2* alterations were largely missense with some truncating mutations, and these were similarly scattered without focal clustering. *ERBB4* and *KMT2A* mutations were predominantly missense and lacked recurrent hotspot patterns.

### 3.4. Mutation Differences by Sex and Race

Mutation enrichment analysis by sex revealed a statistically significant association between male sex and mutations in *FLT3* (n = 61; *p* < 0.001), compared to females (n = 2; *p* < 0.001) after FDR correction (q < 0.05).

Racial stratification analysis identified low-frequency genomic alterations observed in specific demographic groups within this cohort (Table 3). Notably, mutations in *SLC28A3* were identified within the Black patient cohort (n = 1; 16.7%) and were not observed in White, Asian, and other racial populations (*p* < 0.001). Additionally, *RECQL4* alterations demonstrated a distinct pattern of enrichment; while present at low frequency in White patients (0.4%), they were significantly more prevalent in Black patients (16.7%; *p* < 0.001). These findings indicate that while major drivers such as *TP53* are shared across populations, certain low-frequency variants may demonstrate cohort-specific distribution patterns.

Subgroup analyses by sex and race were performed using chi-squared testing. Given the limited sample sizes in certain strata, particularly among Asian (n = 4) and Black patients (n = 6), these comparisons are exploratory and should be interpreted with caution.

### 3.5. Patterns of Co-Alteration and Mutual Exclusivity

Pairwise co-occurrence analysis identified significant associations among frequently mutated genes. Table 4 highlights the highest-confidence associations, while Appendix A Table A3 presents the complete set of statistically significant gene–gene pairs. Significantly co-occurring pairs included *TP53–CDKN2A* (n = 148), *NOTCH1–FAT1* (n = 88), *NOTCH1–KMT2D* (n = 110), *NOTCH1–NOTCH2* (n = 92), *TP53–ROS1* (n = 113), and *ROS1–ERBB4* (n = 59). Additional significant associations included *NOTCH1–CDKN2A* (n = 118), *KMT2D–FAT1* (n = 66), *NOTCH1–ROS1* (n = 88), *KMT2D–ERBB4* (n = 66), *KMT2D–KMT2A* (n = 56), *ROS1–KMT2A* (n = 46), and *TP53–KMT2A* (n = 75) (*p* < 0.001 and q < 0.001 for all comparisons shown in Table 4). Co-occurrence was defined as the number of patients with alterations in both genes divided by the total number of patients with an alteration in either gene, excluding cases in which neither gene was altered.

No gene pairs met the significance threshold for mutual exclusivity after correction.

## 4. Discussion

Our findings align with prior cSCC genomic studies, particularly with respect to recurrent alterations in established drivers such as *TP53*, *NOTCH1*, and *CDKN2A*. What distinguishes the present analysis is the integrated evaluation of mutation frequency, hotspot context, copy-number and LOH events, and statistically tested gene–gene co-occurrence patterns within a single harmonized, multi-institutional dataset. By examining these analytic layers together, this study provides a structured view of how recurrent alterations cluster within broader pathway-level disruption. This integrated framework refines the genomic landscape of cSCC beyond isolated gene frequencies and establishes a transparent, cohort-level reference for future functional and clinically annotated investigations.

### 4.1. Genomic Landscape and Demographic Patterns

Using the AACR Project GENIE consortium, this study characterizes the genomic architecture of cSCC. Comprehensive genomic analysis identified the most commonly altered genes, delineated significant patterns of gene co-occurrence, and detected exploratory demographic-associated variation among select low-frequency alterations. Together, these findings provide new insight into the molecular complexity of cSCC and emphasize the contribution of disrupted genomic integrity, differentiation pathways, epigenetic regulation, and growth signaling to its pathogenesis.

The cohort was predominantly male (75.6%) and White (85.5%), consistent with established epidemiologic patterns indicating a higher incidence among older, fair-skinned men [1,6]. Beyond broad demographic trends, our analysis identified mutational patterns within specific racial subgroups. The identification of *SLC28A3* mutations exclusively in Black patients, alongside significant enrichment of *RECQL4*, raises the possibility of demographic-associated genomic variation within cSCC. *RECQL4* plays a critical role in DNA integrity and helicase activity; its enrichment in Black patients, who are historically less prone to UV-induced carcinogenesis, may reflect alternative pathogenic pathways. However, the biological significance of this observation remains uncertain. These subgroup-specific observations underscore the importance of diverse genomic cohorts, as they may identify alterations that are underrepresented in less diverse datasets. Nonetheless, these subgroup-associated findings should be interpreted with caution. The statistical power of this sub-analysis is severely limited by the small number of patients in the Asian (n = 4) and Black (n = 6) cohorts, making the results susceptible to statistical instability. These associations, while statistically significant after FDR correction, are based on single-event alterations and require validation in larger, more diverse cohorts before any definitive conclusions can be drawn.

### 4.2. Frequently Altered Genes and Pathways

Comprehensive genomic profiling of this cohort revealed substantial genetic heterogeneity, with recurrent mutations affecting pathways involved in genomic stability, keratinocyte differentiation, chromatin regulation, cell-cycle control, telomere maintenance, and receptor tyrosine kinase signaling. The most frequently mutated genes were *TP53* (83.5%), *NOTCH1* (56.3%), *KMT2D* (47.0%), *CDKN2A* (44.4%), *TERT* (41.4%), *ROS1* (34.3%), *FAT1* (33.3%), *NOTCH2* (31.2%), *ERBB4* (28.4%), and *KMT2A* (24.3%). These findings align with prior studies reporting *TP53*, *NOTCH1*, *KMT2D*, *CDKN2A*, *TERT*, *FAT1*, *NOTCH2*, and *ERBB4* as key contributors to cSCC biology [12,14,15,16,17,18]. Notably, *ROS1* and *KMT2A* are not emphasized in cSCC literature despite their relatively high prevalence in our cohort and may merit further investigation as underrecognized contributors to cSCC.

The most frequently mutated genes in this dataset participate in diverse biological functions. *TP53* regulates DNA damage response and apoptosis, serving as a central genomic guardian in keratinocytes exposed to ultraviolet radiation [19]. *NOTCH1* and *NOTCH2* are critical regulators of epidermal differentiation, maintaining stratified epithelial structure and restricting basal-cell proliferation [17]. *KMT2D* and *KMT2A* encode histone methyltransferases that control enhancer activation and transcriptional accessibility [12], while *CDKN2A* mediates G1–S checkpoint enforcement through inhibition of CDK4/6 [16]. *TERT* promotes telomere maintenance and replicative immortality [15]. *ROS1* and *ERBB4* activate receptor tyrosine kinase pathways that enhance proliferation and survival, whereas *FAT1* modulates cell adhesion and Wnt pathway suppression [18]. Collectively, these alterations implicate cooperative impairment of genomic surveillance, differentiation, chromatin remodeling, and growth-factor signaling as core features of cSCC biology.

### 4.3. p53 Pathway

The exceptionally high frequency of *TP53* mutations in this cohort (83.5%) aligns with previous literature reporting this mutation in 81% of cSCC lesions [20]. p53 functions as a critical genomic gatekeeper in keratinocytes, activating transcriptional programs that mediate cell-cycle arrest, apoptosis, and DNA repair in response to ultraviolet (UV)-induced DNA damage. Loss-of-function alterations in *TP53* remove this essential surveillance mechanism, allowing genetically damaged keratinocytes to evade apoptosis and continue proliferating despite an extensive mutational burden. This process facilitates clonal expansion of mutated cells and promotes the accumulation of additional oncogenic events, establishing a permissive genomic environment that accelerates malignant transformation [19].

Hotspot analysis revealed recurrent mutations affecting the DNA-binding domain, particularly at codon R248, including R248W, R248Q, R248G, and R248H substitutions, as well as N247_R248insQ. *TP53* mutations involving the R248 residue have been demonstrated across numerous cancer types to disrupt p53–DNA binding, effectively abolishing transcriptional activation of downstream cell-cycle and apoptotic regulators [21]. The presence of recurrent hotspot patterns suggests selective pressure favoring functional inactivation of p53 rather than random mutational acquisition, consistent with the role of UV radiation as a potent mutagen driving signature C > T transitions within dipyrimidine sites [22].

Clinically, *TP53* mutations in cSCC have been linked to increased tumor aggressiveness, deeper invasion, perineural spread, and higher metastatic potential [14,15,23]. p53 loss correlates with poorer disease-specific survival and may contribute to resistance to treatments such as radiation and chemotherapy. The frequent coexistence of *TP53* alterations with other key mutations, including *CDKN2A*, *NOTCH1*, and *ROS1*, is consistent with its role as a potential early enabling event in pathway disruption [24].

Therapeutically, p53 inactivation creates potential vulnerabilities that may be exploited through targeted strategies, including DNA repair inhibitors, p53-independent apoptotic therapies, and emerging p53 reactivation approaches. These implications underscore the value of genomic profiling to identify patients who may benefit from mechanism-based treatment strategies. However, these therapeutic considerations warrant validation in larger, clinically annotated cohorts.

### 4.4. Cell Cycle Checkpoint Dysregulation

Dysregulation of the G1–S cell-cycle checkpoint emerged as a major pathogenic theme in this cohort, driven predominantly by alterations in *CDKN2A* (44.4%) and recurrent structural losses affecting the *CDKN2A/CDKN2B* locus [20,25]. *CDKN2A* encodes the tumor suppressor p16^INK4a^, a key regulator that restrains cell-cycle progression. Loss-of-function alterations in this gene disable the checkpoint responsible for stopping cell growth in response to DNA damage or oncogenic signals, allowing continued proliferation despite accumulating genetic injury [25].

Hotspot analysis demonstrated recurrent alterations affecting the R58–V59 region of *CDKN2A*, including R58*, R58Efs*88, R58Q, and V59del variants, highlighting a selective pressure toward functional inactivation of p16^INK4a^. Given the central function of p16^INK4a^ in keratinocyte growth control, its disruption provides a strong proliferative advantage and facilitates clonal expansion of aberrant keratinocyte populations [2,25].

Copy number analysis revealed recurrent LOH at the *CDKN2A* and *CDKN2B* loci, consistent with prior genomic studies [20,25]. Amplification of *CCND1* was also observed, indicating that a subset of tumors may further enhance cell-cycle progression through increased cyclin D1 expression. Elevated cyclin D1 activity promotes CDK4/6-driven phosphorylation of RB1, facilitating unchecked transition into S phase. The coexistence of *CDKN2A* loss and *CCND1* amplification in select tumors highlights a selective advantage for dual checkpoint escape and heightened proliferative capacity [20].

These findings are consistent with a model in which cell-cycle deregulation constitutes a core pathogenic axis in cSCC. Clinically, alterations affecting *CDKN2A* have been associated with increased tumor aggressiveness, metastatic potential, and treatment resistance in cSCC, underscoring their relevance to disease progression [2]. From a therapeutic perspective, tumors harboring *CDKN2A* loss or *CCND1* amplification may demonstrate increased sensitivity to CDK4/6 inhibitors, raising the possibility that targeted therapeutic strategies could benefit a subset of patients with profound checkpoint disruption, although clinical validation in cSCC specifically is required.

### 4.5. Notch Signaling

Disruption of Notch signaling emerged as a major molecular feature in this cohort, with frequent alterations in *NOTCH1* (56.3%) and *NOTCH2* (31.2%), consistent with prior studies reporting Notch pathway mutations in up to 75% of cSCC tumors [17]. In stratified epithelium, Notch signaling promotes keratinocyte differentiation and limits basal-cell proliferation, helping maintain normal epidermal structure. Loss-of-function mutations in *NOTCH1* and *NOTCH2* weaken these differentiation programs, enabling expansion of less differentiated keratinocyte populations with increased proliferative capacity, a shift that supports tumor development and progression [26,27]. Experimental studies further support this mechanism, demonstrating that Notch-deficient keratinocytes exhibit enhanced stem-like properties and increased susceptibility to carcinogenesis following ultraviolet exposure [27].

Functional loss of Notch signaling may cooperate with other high-frequency alterations identified in this cohort. The observed co-occurrence between *NOTCH1* and *FAT1* mutations suggests synergistic impairment of differentiation and Wnt pathway regulation. Similarly, co-alteration with *KMT2D* implicates broader effects on transcriptional control. These patterns indicate that Notch inactivation may act alongside epigenetic and proliferative pathways to promote malignant behavior.

### 4.6. Epigenetic Modification

Epigenetic dysregulation represents a major pathogenic component in this cohort, driven by frequent mutations in chromatin-modifying genes such as *KMT2D* (47.0%) and *KMT2A* (24.3%). These genes encode histone methyltransferases that help regulate which genes are turned on or off during keratinocyte differentiation. Loss-of-function alterations can disrupt this regulatory control, leading to abnormal gene expression patterns and increased cellular plasticity, which may allow keratinocyte populations with malignant potential to persist and expand [28].

Functional impairment of epigenetic regulation may cooperate with other high-frequency alterations identified in this cohort. Co-occurrence of *KMT2D* mutations with *NOTCH1* suggests combined impairment of differentiation programs and transcriptional regulation, while overlap with *TP53* alterations may intensify genomic instability. These intersecting mechanisms create a permissive environment for the acquisition and retention of oncogenic alterations.

Emerging evidence suggests that epigenetic dysregulation, including methylation changes, contributes to high-risk tumors, aggressive behavior, and worse survival in cSCC [29]. Therapeutically, these findings raise the possibility of targeting epigenetic vulnerabilities. Preclinical studies demonstrate that inhibition of histone demethylases such as LSD1 increases H3K4 methylation and suppresses cSCC growth, highlighting the therapeutic potential of targeting epigenetic regulators in keratinocyte-derived cancers [30,31].

### 4.7. Telomere Maintenance

Alterations affecting telomere biology were frequently observed in this cohort, with *TERT* mutations present in 41.4% of samples, consistent with reports describing *TERT* alterations in 50% of cSCC cases [32,33]. Telomere maintenance is a critical mechanism enabling continued cellular proliferation, particularly in keratinocytes repeatedly exposed to ultraviolet radiation–induced DNA damage. *TERT* promoter and coding-region alterations can reactivate telomerase expression, preventing telomere shortening and circumventing replicative senescence [34]. This provides malignant keratinocytes with extended proliferative capacity and supports clonal expansion over time. Clinically, *TERT* activation in cSCC has been associated with increased tumor aggressiveness, higher recurrence rates, and invasive cancer, indicating a significant role in disease persistence and progression [35].

### 4.8. RTK/MAPK Signaling

Receptor tyrosine kinase (RTK) and MAPK pathway activation emerged as another major pathogenic axis, with recurrent alterations in *ROS1* (34.3%) and *ERBB4* (28.4%), as well as amplification events involving *EGFR*. RTK signaling regulates keratinocyte proliferation, survival, and migration, and dysregulation of this pathway can enhance oncogenic signaling output [36]. Co-occurrence of *ROS1* and *ERBB4* mutations suggests potential pathway redundancy or synergy in promoting sustained growth signaling. Amplification of *EGFR* further supports increased mitogenic drive [37]. These patterns highlight the potential therapeutic relevance of RTK-directed inhibitors, as EGFR-targeted agents have demonstrated measurable, though modest, clinical activity in advanced cSCC [38].

### 4.9. Wnt Signaling

Alterations in *FAT1* occurred in 33.3% of cases, highlighting a major role for cell–cell adhesion and Wnt pathway regulation in cSCC. *FAT1* functions as a tumor suppressor that helps control β-catenin signaling and maintain epithelial structure. Loss-of-function mutations weaken this regulatory balance, enabling increased Wnt activity, enhanced proliferation, and reduced adhesion within the epidermis [39]. The frequent co-occurrence of *FAT1* mutations with *NOTCH1* suggests combined impairment of differentiation and adhesion pathways, contributing to the dedifferentiated and invasive phenotype observed in more advanced cSCC [12].

### 4.10. Co-Occurrence Patterns

Pairwise co-occurrence analysis identified significant associations among several frequently mutated genes, including *TP53–CDKN2A*, *NOTCH1–FAT1*, *NOTCH1–KMT2D*, *NOTCH1–NOTCH2*, *TP53–ROS1*, and *ROS1–ERBB4*. These statistically significant co-occurrence patterns are consistent with coordinated pathway disruption in cSCC; however, they reflect genomic associations and are not interpreted as direct evidence of mechanistic cooperation in the absence of functional validation [12].

The strong association between *TP53* and *CDKN2A* supports a model in which simultaneous loss of genomic surveillance and cell-cycle control provides substantial proliferative advantage. Similarly, co-alterations involving *NOTCH1*, *KMT2D*, and *FAT1* indicate coordinated impairment of differentiation, chromatin regulation, and adhesion signaling. The co-occurrence of *ROS1* and *ERBB4* highlights potential convergence on RTK/MAPK signaling, suggesting proliferative pathway redundancy. No statistically significant mutually exclusive gene relationships were identified, reinforcing the concept of integrated pathway disruption as a defining feature of cSCC genomic architecture [16].

### 4.11. Targeted Therapeutic Opportunities

While immune checkpoint inhibitors (e.g., cemiplimab, pembrolizumab) represent the standard of care for advanced cSCC, a significant subset of patients are ineligible for or refractory to these agents. Our genomic data highlight two distinct pathways that may warrant exploration as potential salvage strategies. However, it is important to note that these therapeutic considerations are hypothesis-generating and are based on genomic correlations rather than direct treatment-response data within this cohort.

First, the ubiquity of *CDKN2A* loss (44.4%) provides a biologically plausible rationale for the use of CDK4/6 inhibitors, although therapeutic response was not evaluated in this cohort, and clinical implications remain preliminary. The functional loss of p16^INK4a^ leads to unchecked CDK4/6 activity and hyperphosphorylation of the Retinoblastoma (Rb) protein, driving cell-cycle progression [25]. Pharmacologic agents such as palbociclib, ribociclib, and abemaciclib can restore the G1-S checkpoint in p16-deficient tumors. Although these agents are primarily approved for breast cancer, early clinical data suggests they may have utility in squamous cell carcinomas harboring *CDKN2A* alterations, potentially offering a non-immunotherapy option for nearly half of the cSCC population [40].

Second, our identification of *EGFR* amplifications (3.7%) and *ERBB4* mutations (28.4%) is consistent with prior evidence supporting EGFR-targeted therapy. While cetuximab is currently utilized in cSCC, response rates are variable [1]. *EGFR* copy number amplification represents a biologically plausible candidate biomarker that warrants evaluation in clinically annotated cSCC cohorts to determine whether it correlates with therapeutic response. Furthermore, the presence of *ERBB4* alterations points to the potential utility of pan-HER inhibitors (e.g., afatinib or dacomitinib) that target multiple members of the ErbB family, potentially overcoming resistance mechanisms seen with selective EGFR inhibition [40].

### 4.12. Limitations

This analysis has several limitations that warrant consideration when interpreting the results. First, although the cohort size for cSCC is large, select subgroup analyses, particularly those stratified by race, sex, or pediatric status, remain limited in statistical power due to the small representation of minority and pediatric patients. As a result, demographic enrichment results should be interpreted cautiously and confirmed in larger, more demographically diverse cohorts. Second, because the dataset is cross-sectional and lacks longitudinal sampling, we are unable to evaluate temporal changes in the mutational landscape over the course of disease progression or therapeutic exposure. As a result, it is not possible to reliably differentiate early driver events from alterations that may arise later in tumor evolution. Third, sequencing heterogeneity across participating institutions may introduce technical bias in mutation frequency estimates. The AACR GENIE dataset aggregates data generated using multiple targeted sequencing platforms with variable gene coverage, reporting thresholds, and assay sensitivity. As a result, not all genes were assessed uniformly across the cohort, and genes included only on larger panels may appear underrepresented. While the GENIE harmonization pipeline mitigates many of these issues, residual batch effects and platform-related variability cannot be entirely ruled out. Fourth, the GENIE database does not include transcriptomic data, which restricts our ability to assess gene expression dynamics or downstream pathway activity. This is particularly relevant in cSCC, where pathway dysregulation may occur through non-mutational mechanisms such as altered gene expression or signaling pathway activation independent of genetic alteration. The absence of transcriptomic and microRNA data also restricts investigation into regulatory mechanisms that may influence tumor behavior or therapeutic response. Fifth, detailed clinical information, including disease stage, histologic subtype, survival outcomes, and treatment history, is not available for this cohort. As a result, we cannot assess the prognostic or predictive significance of the observed mutations, nor determine how specific genomic alterations relate to therapy response or resistance. Without these data, potential associations between genomic profiles and clinical course remain unexplored. Sixth, although the GENIE initiative attempts to minimize duplicate sampling, the inclusion of multiple tumor specimens from an individual patient cannot be entirely excluded, particularly in studies involving both primary and metastatic lesions. This may skew mutation frequencies or overrepresent alterations associated with advanced disease. Seventh, the analysis aggregates all cSCC cases without stratification by histologic subtype or disease stage, limiting the ability to identify subtype-specific genomic patterns or their clinical implications. Finally, because sequencing was performed on tumor-only specimens, germline variants cannot be definitively excluded. Although common polymorphisms are filtered through the GENIE harmonization pipeline, rare pathogenic germline variants may remain, potentially leading to an overestimation of true somatic mutation frequency. Alterations with high variant allele fractions should therefore be interpreted with caution until validated through matched tumor-normal sequencing.

Despite these limitations, this study represents one of the most extensive genomic analyses of cSCC to date and provides a valuable foundation for future mechanistic and translational investigations.

## 5. Conclusions

This large-scale genomic analysis of cSCC reveals recurrent alterations affecting genomic integrity, differentiation, epigenetic regulation, cell-cycle control, telomere maintenance, and growth-factor signaling. Frequently altered genes included *TP53*, *NOTCH1*, *KMT2D*, *CDKN2A*, and *TERT*, accompanied by recurrent copy number losses in *CDKN2A/CDKN2B* and amplifications in *CCND1* and *EGFR*, underscoring the coordinated disruption of key regulatory pathways driving cSCC development and progression. Patterns of gene co-alteration further support cooperative pathway impairment, suggesting that malignant transformation arises through integrated rather than isolated genomic events.

By delineating the molecular landscape of cSCC across a large, multi-institutional cohort, this study enhances current understanding of the genomic architecture underlying this common yet genomically understudied malignancy. These findings provide a framework for future investigations aimed at validating pathway-specific mechanisms, evaluating subgroup-associated genomic patterns in larger and more diverse cohorts, and exploring targeted diagnostic and therapeutic strategies for patients with cSCC.

## Figures and Tables

**Table 1:** cSCC patient demographics (N = 423 samples; 406 patients).

Demographics	Category	N (%)
Sex	Male	307 (75.6)
	Female	86 (21.2)
	Unknown	13 (3.2)
Age Category	Adult	405 (99.8)
	Pediatric	1 (0.2)
Ethnicity	Non-Hispanic	330 (81.3)
	Unknown/Not Collected	66 (16.3)
	Hispanic	10 (2.5)
Race	White	347 (85.5)
	Black	6 (1.5)
	Asian	4 (1.0)
	Other	9 (2.2)
	Unknown/Not Collected	40 (9.9)
Sample Type	Primary	226 (53.4)
	Metastasis	180 (42.6)
	Unspecified/Not Collected	17 (4.0)

**Table 2:** Mutation frequency in cSCC.

Gene	N (%)
*TP53*	353 (83.5)
*NOTCH1*	238 (56.3)
*KMT2D*	199 (47.0)
*CDKN2A*	188 (44.4)
*TERT*	175 (41.4)
*ROS1*	145 (34.3)
*FAT1*	141 (33.3)
*NOTCH2*	132 (31.2)
*ERBB4*	120 (28.4)
*KMT2A*	103 (24.3)

**Table 3:** Somatic mutation enrichment by race following FDR correction.

Gene (Chi-Squared)	Asian, N (%)	Black, N (%)	White, N (%)	*p* Value
*SLC28A3*	0 (0.00)	1 (16.67)	0 (0.00)	*p* < 0.001
*RECQL4*	0 (0.00)	1 (16.67)	1 (0.40)	*p* < 0.001

**Table 4:** Significant co-occurring mutations in cSCC.

A	B	N (%)	95% CI	Log2 Odds Ratio	*p* Value	Q Value
*TP53*	*CDKN2A*	148 (57.8)	51.5–63.9%	2.520	*p* < 0.001	q < 0.001
*NOTCH1*	*FAT1*	88 (55.0)	46.9–62.9%	2.802	*p* < 0.001	q < 0.001
*NOTCH1*	*CDKN2A*	118 (54.1)	47.3–60.9%	1.789	*p* < 0.001	q < 0.001
*NOTCH1*	*KMT2D*	110 (50.7)	43.8–57.5%	1.533	*p* < 0.001	q < 0.001
*NOTCH1*	*NOTCH2*	92 (45.5)	38.5–52.7%	1.949	*p* < 0.001	q < 0.001
*TP53*	*ROS1*	113 (45.4)	39.1–51.8%	3.964	*p* < 0.001	q < 0.001
*KMT2D*	*FAT1*	66 (43.1)	35.2–51.4%	1.467	*p* < 0.001	q < 0.001
*NOTCH1*	*ROS1*	88 (42.7)	35.9–49.8%	1.519	*p* < 0.001	q < 0.001
*ROS1*	*ERBB4*	59 (39.6)	31.7–47.9%	2.086	*p* < 0.001	q < 0.001
*KMT2D*	*ERBB4*	66 (37.7)	30.5–45.3%	1.726	*p* < 0.001	q < 0.001
*KMT2D*	*KMT2A*	56 (33.3)	26.3–41.0%	1.845	*p* < 0.001	q < 0.001
*ROS1*	*KMT2A*	46 (31.7)	24.3–40.0%	1.648	*p* < 0.001	q < 0.001
*TP53*	*KMT2A*	75 (30.1)	24.5–36.2%	4.044	*p* < 0.001	q < 0.001

Co-occurrence percentages were calculated as the number of patients harboring alterations in both genes divided by the total number of patients with an alteration in at least one of the two genes (i.e., A only, B only, or both). Patients without alterations in either gene were excluded from the denominator.

## Data Availability

The genomic dataset analyzed in this study is available through the AACR Project GENIE consortium via the cBioPortal platform (https://genie.cbioportal.org/). Accessed 22 November 2025.

## Abbreviations

The following abbreviations are used in this manuscript:

AACR	American Association for Cancer Research
cSCC	Cutaneous Squamous Cell Carcinoma
CI	Confidence Interval
CNA	Copy Number Alteration
*CDKN2A*	Cyclin Dependent Kinase Inhibitor 2A
*CDKN2B*	Cyclin Dependent Kinase Inhibitor 2B
DNA	Deoxyribonucleic Acid
*EGFR*	Epidermal Growth Factor Receptor
*ERBB4*	Erb-B2 Receptor Tyrosine Kinase 4
*FAT1*	FAT Atypical Cadherin 1
*FLT3*	Fms-Like Tyrosine Kinase 3
FDR	False Discovery Rate
GENIE	Genomics, Evidence, Neoplasia, Information, Exchange
LOH	Loss of Heterozygosity
MAPK	Mitogen-Activated Protein Kinase
MSK	Memorial Sloan Kettering
NGS	Next-Generation Sequencing
*NOTCH1*	Neurogenic Locus Notch Homolog Protein 1
*NOTCH2*	Neurogenic Locus Notch Homolog Protein 2
RTK	Receptor Tyrosine Kinase
*RECQL4*	RecQ Like Helicase 4
*ROS1*	ROS Proto-Oncogene 1
SD	Standard Deviation
*SLC28A3*	Solute Carrier Family 28 Member 3
TERT	Telomerase Reverse Transcriptase
TP53	Tumor Protein p53
UV	Ultraviolet Radiation
VAF	Variant Allele Frequency

## Appendix A

**Table A1:** Cohort composition by Center (N = 423 samples; 406 patients).

Center	Samples (%)	Patients (%)
MSK	173 (40.9)	166 (40.9)
DFCI	93 (22.0)	92 (22.7)
UCSF	59 (13.9)	54 (13.3)
WAKE	22 (5.2)	22 (5.4)
NKI	14 (3.3)	11 (2.7)
VICC	13 (3.1)	12 (3.0)
MDA	12 (2.8)	12 (3.0)
DUKE	8 (1.9)	8 (2.0)
PROV	6 (1.4)	6 (1.5)
UHN	6 (1.4)	6 (1.5)
COLU	4 (0.9)	4 (1.0)
GRCC	4 (0.9)	4 (1.0)
SCI	3 (0.7)	3 (0.7)
JHU	2 (0.5)	2 (0.5)
VHIO	2 (0.5)	2 (0.5)
YALE	2 (0.5)	2 (0.5)

**Table A2:** Cohort composition by Sequence Assay ID (N = 423 samples; 406 patients).

Sequence Assay ID	Samples (%)	Patients (%)
MSK-IMPACT468	66 (15.6)	63 (15.5)
MSK-IMPACT505	46 (10.9)	45 (11.1)
MSK-IMPACT410	43 (10.2)	42 (10.3)
DFCI-ONCOPANEL-3.1	32 (7.6)	32 (7.9)
DFCI-ONCOPANEL-2	29 (6.9)	29 (7.1)
UCSF-IDTV5-TO	26 (6.1)	25 (6.2)
UCSF-NIMV4-TN	19 (4.5)	15 (3.7)
MSK-IMPACT341	18 (4.3)	18 (4.4)
WAKE-CLINICAL-DX1	18 (4.3)	18 (4.4)
DFCI-ONCOPANEL-1	15 (3.5)	15 (3.7)
DFCI-ONCOPANEL-3	15 (3.5)	15 (3.7)
NKI-CHPV2-SOCV2-NGS	11 (2.6)	9 (2.2)
VICC-01-T7	10 (2.4)	9 (2.2)
UCSF-NIMV4-TO	9 (2.1)	9 (2.2)
DUKE-F1-DX1	7 (1.7)	7 (1.7)
MDA-50-V1	6 (1.4)	6 (1.5)
PROV-TSO500HT-V2	5 (1.2)	5 (1.2)
UCSF-IDTV5-TN	5 (1.2)	5 (1.2)
COLU-CCCP-V1	4 (0.9)	4 (1.0)
GRCC-MOSC3	3 (0.7)	3 (0.7)
NKI-TSACP-MISEQ-NGS	3 (0.7)	2 (0.5)
SCI-PMP68-V1	3 (0.7)	3 (0.7)
DFCI-F1-AB1	2 (0.5)	2 (0.5)
JHU-50GP	2 (0.5)	2 (0.5)
MDA-409-V1	2 (0.5)	2 (0.5)
MDA-46-V1	2 (0.5)	2 (0.5)
UHN-OCA-V3	2 (0.5)	2 (0.5)
UHN-TSO500-V1	2 (0.5)	2 (0.5)
VICC-01-T5A	2 (0.5)	2 (0.5)
WAKE-CLINICAL-DX2	2 (0.5)	2 (0.5)
YALE-OCP-V3	2 (0.5)	2 (0.5)
DUKE-F1-T7	1 (0.2)	1 (0.2)
GRCC-OCAV3	1 (0.2)	1 (0.2)
PROV-FOUNDATIONONELIQUIDCDX	1 (0.2)	1 (0.2)
UHN-48-V1	1 (0.2)	1 (0.2)
UHN-555-V1	1 (0.2)	1 (0.2)
VICC-01-T4B	1 (0.2)	1 (0.2)
VHIO-300	1 (0.2)	1 (0.2)
VHIO-SKIN-V01	1 (0.2)	1 (0.2)
WAKE-CA-NGSQ3	1 (0.2)	1 (0.2)
WAKE-CLINICAL-T7	1 (0.2)	1 (0.2)

**Table A3:** Complete significant co-occurrence results.

A	B	N (%)	95% CI	Log2 Odds Ratio	*p* Value	Q Value
*TP53*	*CDKN2A*	148 (57.8)	51.5–63.9%	2.520	*p* < 0.001	q < 0.001
*NOTCH1*	*FAT1*	88 (55.0)	46.9–62.9%	2.802	*p* < 0.001	q < 0.001
*NOTCH1*	*CDKN2A*	118 (54.1)	47.3–60.9%	1.789	*p* < 0.001	q < 0.001
*NOTCH1*	*KMT2D*	110 (50.7)	43.8–57.5%	1.533	*p* < 0.001	q < 0.001
*NOTCH1*	*NOTCH2*	92 (45.5)	38.5–52.7%	1.949	*p* < 0.001	q < 0.001
*TP53*	*ROS1*	113 (45.4)	39.1–51.8%	3.964	*p* < 0.001	q < 0.001
*KMT2D*	*FAT1*	66 (43.1)	35.2–51.4%	1.467	*p* < 0.001	q < 0.001
*NOTCH1*	*ROS1*	88 (42.7)	35.9–49.8%	1.519	*p* < 0.001	q < 0.001
*ROS1*	*ERBB4*	59 (39.6)	31.7–47.9%	2.086	*p* < 0.001	q < 0.001
*KMT2D*	*ERBB4*	66 (37.7)	30.5–45.3%	1.726	*p* < 0.001	q < 0.001
*KMT2D*	*KMT2A*	56 (33.3)	26.3–41.0%	1.845	*p* < 0.001	q < 0.001
*ROS1*	*KMT2A*	46 (31.7)	24.3–40.0%	1.648	*p* < 0.001	q < 0.001
*TP53*	*KMT2A*	75 (30.1)	24.5–36.2%	4.044	*p* < 0.001	q < 0.001
*TERT*	*FAT1*	61 (42.1)	33.9–50.5%	1.394	*p* < 0.001	q = 0.002
*TP53*	*ERBB4*	90 (35.7)	29.8–42.0%	2.317	*p* < 0.001	q = 0.002
*NOTCH1*	*KMT2A*	60 (30.6)	24.2–37.6%	1.508	*p* < 0.001	q = 0.002
*NOTCH1*	*TERT*	85 (42.7)	35.7–49.9%	1.299	*p* = 0.001	q = 0.003
*NOTCH2*	*KMT2A*	43 (29.1)	21.9–37.1%	1.320	*p* < 0.001	q = 0.003
*TP53*	*FAT1*	98 (47.6)	40.6–54.6%	2.001	*p* = 0.002	q = 0.004
*CDKN2A*	*FAT1*	67 (42.1)	34.4–50.2%	1.245	*p* = 0.002	q = 0.004
*CDKN2A*	*TERT*	73 (39.9)	32.7–47.4%	1.147	*p* = 0.002	q = 0.004
*KMT2D*	*ROS1*	73 (38.4)	31.5–45.7%	1.154	*p* = 0.002	q = 0.004
*NOTCH1*	*ERBB4*	71 (35.0)	28.4–42.0%	1.250	*p* = 0.002	q = 0.004
*CDKN2A*	*ROS1*	75 (38.5)	31.6–45.7%	1.093	*p* = 0.002	q = 0.005
*ERBB4*	*KMT2A*	36 (26.9)	19.6–35.2%	1.247	*p* = 0.003	q = 0.005
*FAT1*	*ERBB4*	41 (31.3)	23.5–40.0%	1.269	*p* = 0.004	q = 0.006
*TP53*	*NOTCH1*	164 (62.1)	56.0–68.0%	1.489	*p* = 0.004	q = 0.007
*TP53*	*KMT2D*	136 (52.5)	46.2–58.7%	1.463	*p* = 0.006	q = 0.008
*CDKN2A*	*NOTCH2*	74 (37.8)	30.9–44.9%	1.004	*p* = 0.005	q = 0.008
*FAT1*	*NOTCH2*	53 (36.6)	28.7–44.9%	1.124	*p* = 0.005	q = 0.008
*ROS1*	*FAT1*	51 (35.7)	27.8–44.1%	1.119	*p* = 0.007	q = 0.009
*FAT1*	*KMT2A*	36 (28.3)	20.7–37.0%	1.259	*p* = 0.006	q = 0.009
*NOTCH2*	*ERBB4*	46 (28.4)	21.6–36.0%	0.815	*p* = 0.029	q = 0.040
*ROS1*	*NOTCH2*	55 (31.4)	24.6–38.9%	0.762	*p* = 0.037	q = 0.049
